# Rapid character scoring and tabulation of large leaf‐image libraries using Adobe Bridge

**DOI:** 10.1002/aps3.11500

**Published:** 2022-11-24

**Authors:** Gabriella Rossetto‐Harris, Elena Stiles, Peter Wilf, Michael P. Donovan, Xiaoyu Zou

**Affiliations:** ^1^ Department of Geosciences, Pennsylvania State University University Park Pennsylvania 16802 USA; ^2^ Department of Biology University of Washington Seattle Washington 98105 USA; ^3^ Department of Paleobotany and Paleoecology Cleveland Museum of Natural History Cleveland Ohio 44106 USA; ^4^ Institute of Geophysics and Planetary Physics, Scripps Institution of Oceanography University of California San Diego La Jolla California 92093 USA

**Keywords:** character data, digital image library, insect damage, leaf architecture, leaves, paleobotany

## Abstract

**Premise:**

Digital image libraries are an integral part of specimen‐based research. However, coding and extracting metadata for hundreds of specimens on a personal computer can be complex. In addition, most existing workflows require downsampling or platform switching and do not link character data directly to the images.

**Methods and Results:**

We demonstrate a method to code and embed into images the standard leaf architecture and insect‐damage characters that are widely used in paleobotany. Using the visual file browser Adobe Bridge, customizable and searchable keywords can be applied directly and reversibly to individual full‐resolution images, and the data can be extracted and formatted into a matrix using scripts.

**Conclusions:**

Our approach is intuitive and acts as a digital mimic and complement to the experience of sorting and analyzing specimens in‐person. Keywords can be easily customized for other data types that require visual sorting using image libraries.

Researchers frequently classify, compare, and analyze thousands of extant and fossil specimens from multiple institutions worldwide to complete systematic and paleoecological research. Photographing collections during museum and herbaria visits, downloading images from online collections, and consolidating the resulting digital image library on a personal computer drastically reduces the time and funding required for travel by allowing remote specimen work. Digital collections have been especially valuable during the COVID‐19 pandemic's significant travel and access restrictions, allowing research to continue from any location. Thus, researchers require efficient workflows for managing their images and associated data on personal computers. They may also wish to develop and contribute their image and specimen data to the public domain on open databases such as Integrated Digitized Biocollections (iDigBio; https://www.idigbio.org [accessed 5 October 2021]), Global Biodiversity Information Facility (GBIF; https://www.gbif.org [accessed 12 December 2021]), and the upcoming EarthCube Paleobotany Database (PBOT) project (Currano et al., 2021; https://pbotportal.weebly.com/ [accessed 17 October 2022]). Widely used programs to manage specimen data, such as Microsoft Excel and Microsoft Access, do not interact directly with images of specimens. The commonly used FilemakerPro software (i.e., Ash et al., 1999; Wing et al., 2009; Currano et al., 2010; Carvalho et al., 2021) integrates character data and images into a single layout but requires that images be individually downsampled and copied into a different platform, making it impractical to attach metadata to large image libraries. In addition, formatting the morphological character data into a matrix for statistical and phylogenetic analyses or species descriptions is particularly time consuming and non‐intuitive across the various platforms.

Here, we present a user‐friendly approach to integrate character data with image libraries, applied to the standard paleobotanical methodologies related to the inventory and description of fossil leaf floras and their insect‐feeding damage. Isolated angiosperm leaves are among the most abundant plant macrofossils, each rich with informative shape and venation (leaf architecture) characters. When working with large fossil‐leaf collections, an important preliminary task is sorting them into possible species groups (often called morphotypes) based on standardized leaf architectural features (Hill, 1982; Johnson et al., 1989; Ash et al., 1999; Ellis et al., 2009; Contreras, 2018). Whole‐flora analyses using fossil leaf morphotypes yield critical paleoecological information and provide a descriptive foundation for systematic studies. However, the task of preliminary morphotyping of a fossil flora on a computer screen from specimen images creates a unique challenge, wherein viewing leaves side by side for comparison, scoring leaves for character states, storing the data, and ultimately categorizing all the specimens can be cumbersome. Similarly, distinctive insect‐feeding damage types on fossil leaves are commonly used to study the evolution and ecology of plant–insect associations through geologic time (i.e., Currano et al., 2010; Donovan et al., 2014, 2020). Insect‐feeding damage on compressed fossil plants has been standardized into a system of discrete damage types based on shared morphological characteristics (Labandeira et al., 2007). Damage types also can be assigned using image libraries of fossil plants, concurrently with morphotyping, and there is a need to rapidly categorize and compare insect damage across fossil and extant host plants and floras (Wilf et al., 2017; Donovan et al., 2020).

Over many years of testing different platforms, we find that Adobe Bridge, an open‐access file management software application and visual browser, is an ideal research tool with customizable features that can meet the needs of plant scientists who need to rapidly assign character data to large libraries of specimen images. Bridge provides a platform to view, annotate, and search image libraries directly and interactively, without any need to duplicate, transfer, or downsample files. Keywords in Bridge are user‐defined and are assigned to images using a simple checkbox format, rapidly creating extractable metadata within each full‐resolution image file. We have used Bridge keywords to annotate fossil plant and herbarium images with their informative morphological features (i.e., Wilf et al., 2009, 2017, 2022; Rossetto‐Harris et al., 2020; Zou, 2021) and insect damage types (i.e., Donovan et al., 2014, 2020). Other previous studies illustrated the utility of Bridge keywording for the digital curation of cultural image collections (Reamer, 2009; Frisch, 2012), as a research tool to quantify behavioral ecological data from photographs (Worley, 2019), and to append locality metadata to fossil specimen images during the digitization process (Contreras, 2018).

We demonstrate how the paleobotany community can use Adobe Bridge as an image‐based research workspace on a personal computer, using keywording to facilitate fossil leaf categorization and to record insect damage types directly on digital images. We provide a metadata dictionary file for import into Bridge, containing a standardized set of leaf architectural and damage type keywords (following Labandeira et al., 2007; Ellis et al., 2009), and we present accompanying code to export and format the keyworded character data into matrices for other analyses and contributions to online databases. An illustration summarizing the workflow presented here (as detailed in Appendix 1) is shown in Figure 1. Our fossil leaf keyword set can be used as a model and customized for any type of digital image library.

**Figure 1:**
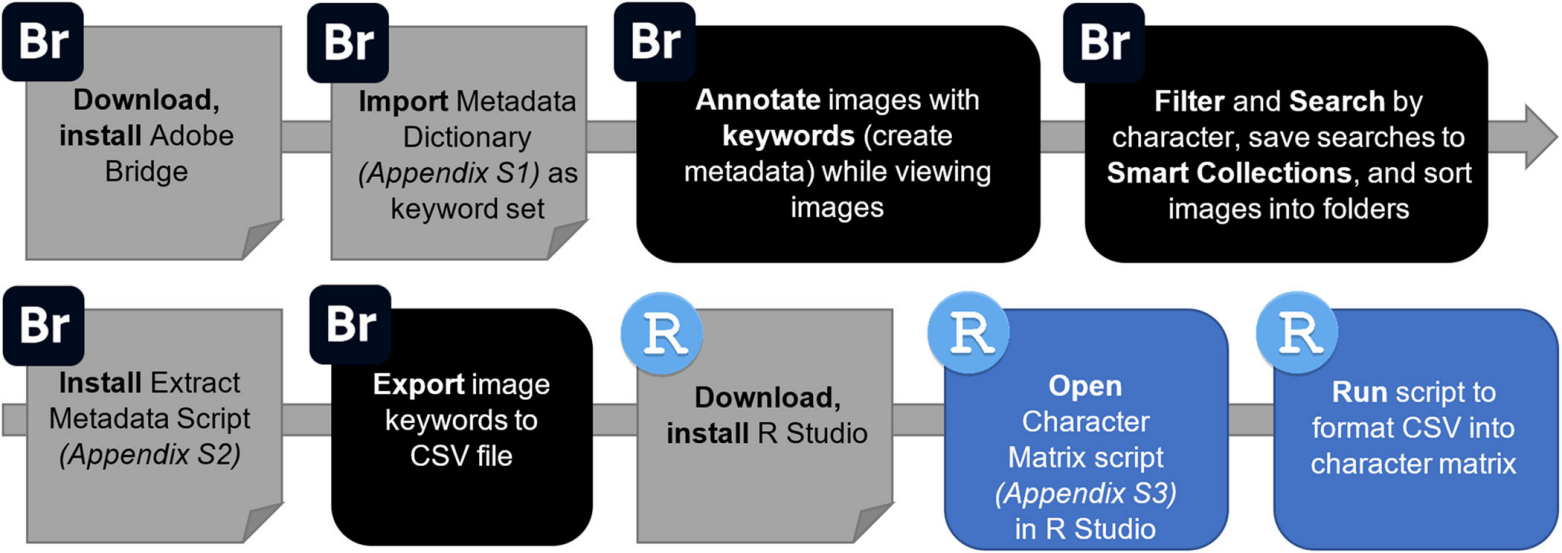
Flowchart illustrating the workflow to annotate large image libraries with character data and create character matrices; the flowchart summarizes the major steps outlined in Appendix 1. The icons at the top left of each box indicate which program is used for that step in the process, either Adobe Bridge (Br) or RStudio (R). Gray boxes denote initial setup tasks, and black and blue boxes represent iterative tasks in Bridge and RStudio, respectively.

## METHODS AND RESULTS

### Paleobotany metadata dictionary

Adobe Bridge (here using version 11.1.1; Adobe Inc., San Jose, California, USA) is a desktop visual file browser and management application. Importantly, Bridge is freely available, accessible as a standalone product without a paid subscription to the full Adobe Creative Cloud software suite (Appendix 1, part I). Adobe Bridge offers a Keywords panel to apply hierarchical, user‐defined labels to images as metadata tags ascribed to each image file. We have found that applying a set of uniform image tags across localities, collections, and researchers allows the user to search, compare, and study specimen images efficiently and effectively.

**Figure 2:**
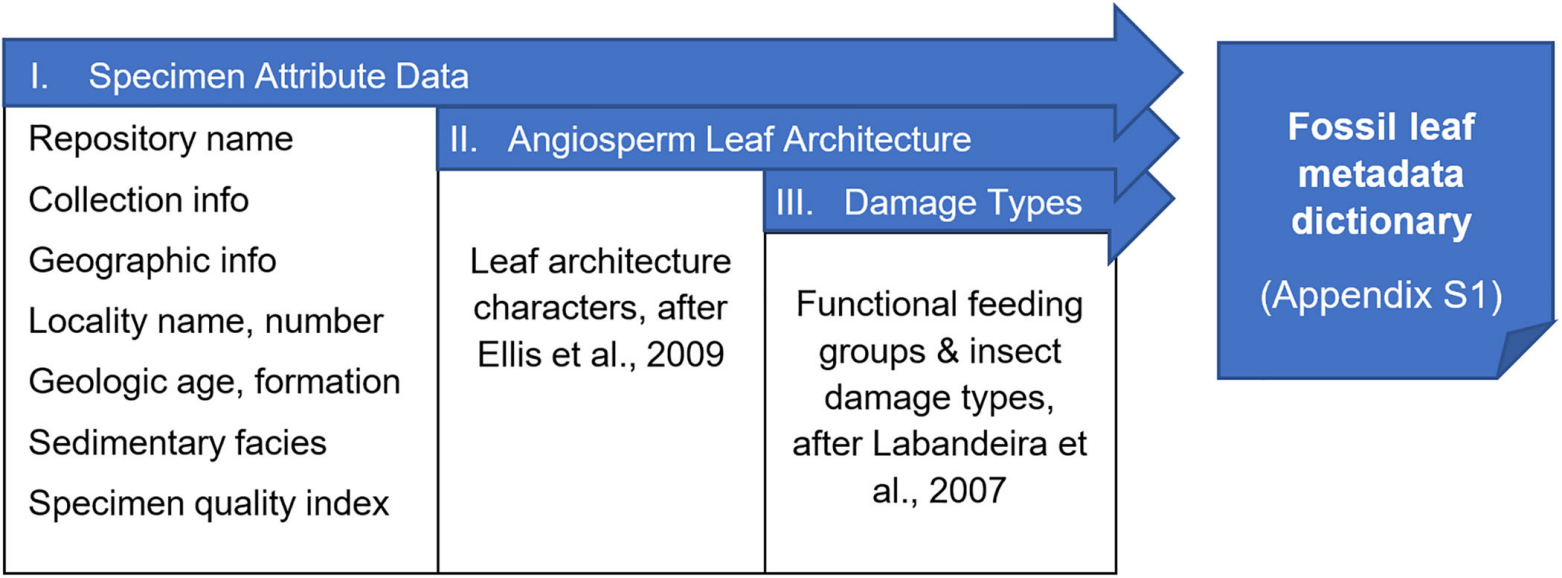
Illustration of the components of the fossil leaf metadata dictionary (Appendix S1), which is specific to the study of fossil leaf‐image libraries. This example can be used as a template for other types of image libraries and their respective character sets.

We provide a metadata dictionary (Appendix S1) accounting for three major categories of data frequently applied to fossil leaf specimens by paleobotanists: specimen attributes, leaf architecture, and damage types (Figure 2). Specimen attributes are fields for standard collections information such as fossil site and repository, among many others. We provide some generic examples, but any customized attributes can be created for multiple collections and projects. For leaf architecture, for ease of comparison we mostly use the same hierarchical numbering system of characters and character states presented in the *Manual of Leaf Architecture* (Ellis et al., 2009; i.e., character numbers 1–52 for shape, venation, and tooth characters). Character numbers 53–63 follow the functional feeding groups and respective damage types (DTs) defined in the *Guide to Insect (and Other) Damage Types on Compressed Plant Fossils* (Labandeira et al., 2007). The damage type keywords include the damage type number and a brief description. Full descriptions and photographs of each damage type are available in Labandeira et al. (2007). The complete keyword text file in Appendix S1 can be uploaded directly into Bridge without changes (Appendix 1, part II) or edited in any text editor and used as a template to create a customized, importable keyword set for other projects.

### Using Bridge keywords

Keywording is an intuitive process of checking user‐defined boxes that appear under the Keyword tab in Bridge while directly viewing a selected image (Appendix 1, part II). Almost any type of image file can be keyworded in Bridge without conversion or other image alteration (the keywords are automatically stored in sidecar files managed by Bridge), including native raw formats for nearly all camera models. Users can easily annotate any number of images simultaneously via group selection. Once images are keyworded, the user can easily filter, search, organize, visually compare, and cross‐validate extensive collections of images using any combination of characters (Appendix 1, part III). Searches can be saved as one‐click Smart Collections that will automatically update as keywords are edited across libraries. Bridge can search for keywords within a single folder, across many subfolders, or even across the entire computer.

These functionalities in Bridge are particularly useful in the morphotyping process, for example, using a specimen quality index to sort the collections for preliminary identification of the best material, or to instantly view specimens with distinctive character combinations across directories to determine whether they lie within the range of variation of a single biologic entity. This method also allows searching and direct visual comparisons of fossils with images of cleared leaves, herbarium sheets, or live‐plant photographs scored for the same characters, thus creating a workflow that would be impractical to accomplish with physical specimens, especially from multiple institutions. While viewing and coding images within Bridge, it is straightforward to move them in and out of folders to sort similar specimens into or out of a “pile” or a filtered group of exemplars, just as one would do in the physical museum space. Keyworded libraries can be shared within or among workgroups using standard cloud storage platforms that synchronize to local devices (e.g., Dropbox, Microsoft OneDrive).

### Exporting character matrices

Keyword metadata can be extracted from images and converted to a character matrix, if desired, using the code provided in Appendices S2 and S3. First, the addition of an open source code by Paul Riggott adds an “Extract Metadata” tool to Bridge (reproduced with permission in Appendix S2; see Appendix 1, part IV). This tool exports a comma‐separated values (CSV) file of all the keywords assigned to a set of selected images. Next, code that we developed in the open source software RStudio (R Core Team, 2021) reformats the aforementioned CSV file into separate attribute, leaf architecture, and insect‐damage character matrices, with rows for each image selected and columns for each character (Appendix 1, part V). The output character matrices are ready for final adjustments for data analyses and preparation for external online databases.

### Tests of the method

We have used and developed the general Bridge keywording methods discussed here to investigate large fossil and extant plant‐image libraries for more than a decade, consolidated from in‐person and virtual visits to collections held at institutions in several countries (i.e., Wilf et al., 2009, 2017; Donovan et al., 2017, 2020; Rossetto‐Harris et al., 2020). The use of keywords to codify characters of leaves (and reproductive structures) has aided us, for example, in the rapid differentiation of fossil conifer species and comparisons with their living relatives and helped us to efficiently classify and compare insect damage types across fossil and extant plants. We subsequently found that leveraging the hierarchical nature of keywords to explicitly follow the shape, vein, and tooth characters from the *Manual of Leaf Architecture* (Ellis et al., 2009) was an effective way to sort and classify several new fossil leaf species present in Neogene floras from Brunei (Zou, 2021; Wilf et al., 2022). Currently, the lead author is using Adobe Bridge to manage image metadata, export it into a character matrix, and develop a revised species list for the highly diverse Río Pichileufú macroflora from the Eocene of Patagonia (Berry, 1938; Rossetto‐Harris et al., 2020).

## CONCLUSIONS

Using keywords in Adobe Bridge is a comprehensive and efficient way to view, sort, and assign character data to large fossil image libraries using a convenient personal computer. This method works directly with all or any subset of the specimen images desired, not just those of a few exemplar specimens, without losing resolution, duplicating, or transferring images across platforms or servers, all within a standard file directory on a personal computer. Our workflow mimics the in‐person experience of sorting, inspecting, and flagging specimens in museum drawers. We find that it drastically reduces the in‐person collections time needed while expediting comparisons of collections from different museums and of fossil and modern comparative material. This method also helps to standardize the maintenance, use, and transfer of image libraries and their metadata among research groups. The character data embedded in the images can be exported and reformatted into a matrix using scripts that we provide using the open source software RStudio, and subsequently used for analysis, publication, and further preparation for online databases. Our method can be customized, by creating a keyword set in a text file for Bridge or directly in the Bridge Keywords panel, for image libraries of any type, including digital collections of herbarium specimens, living plant collections, fossil or modern reproductive structures, pollen, cuticle, phytoliths, wood, or fungi. Because Adobe Bridge is available for free download, with access not dependent on Creative Cloud subscription status, and is available in 24 languages, this method is accessible to plant scientists and other users around the globe.

## AUTHOR CONTRIBUTIONS

As discussed in the manuscript, all the authors have contributed over several years to developing, testing, and applying the workflow presented here in several paleobotanical research projects. E.S. wrote the R code with input from G.R.‐H. P.W. and G.R.‐H. implemented the metadata export workflow. G.R.‐H. and M.P.D. created the keyword data dictionary file. G.R.‐H. drafted the manuscript, and all authors provided comments and revisions. All authors approved the final version of the manuscript.

## Supporting information


**Appendix S1**. Fossil leaf metadata dictionary.

**Appendix S2**. Extract metadata script.

**Appendix S3**. Format character matrix script.

## Figures and Tables

**Figure A1:**
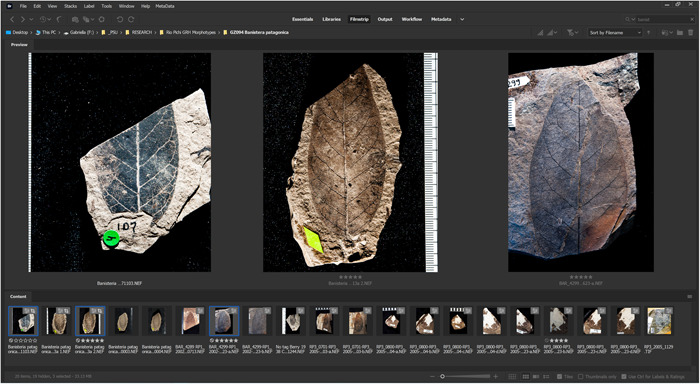
How to view images side by side.

**Figure A2:**
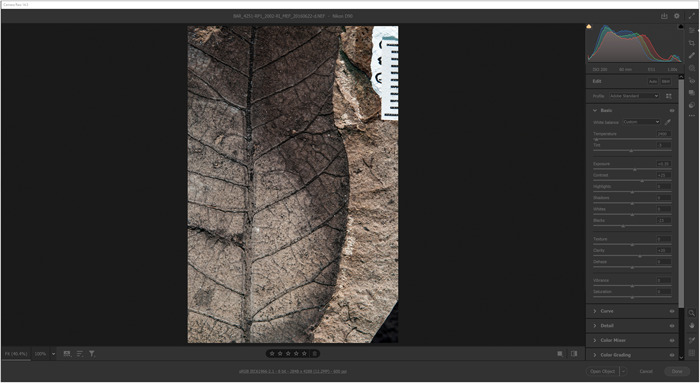
Example of Camera Raw editor.

**Figure A3:**
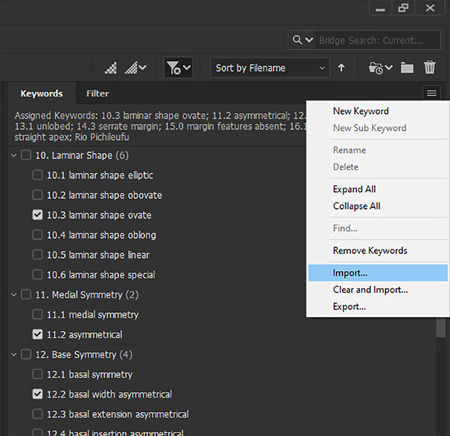
How to import a keyword text file.

**Figure A4:**
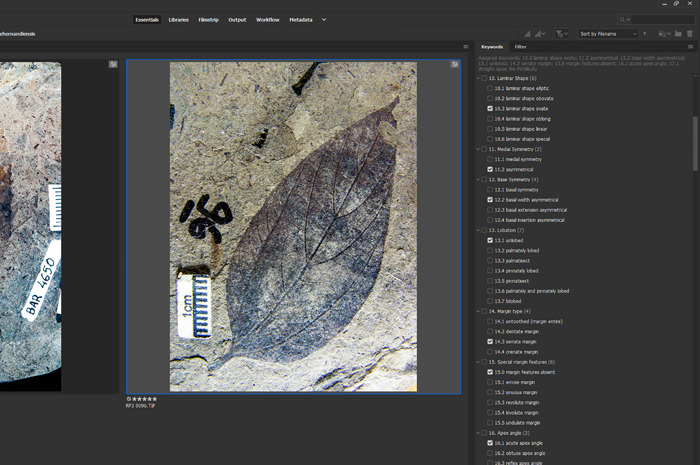
Keywording an image.

**Figure A5:**
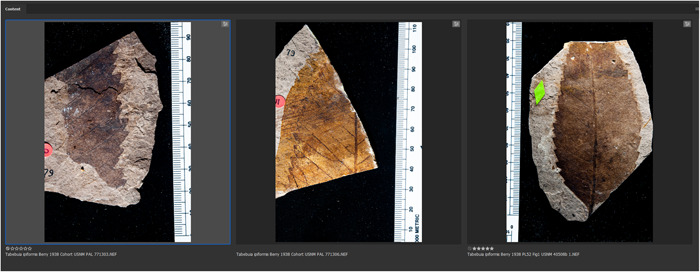
Image rating example.

**Figure A6:**
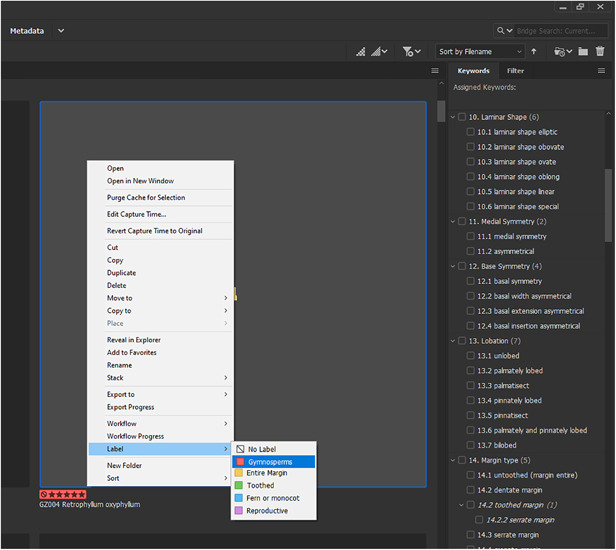
Example of using labels.

**Figure A7:**
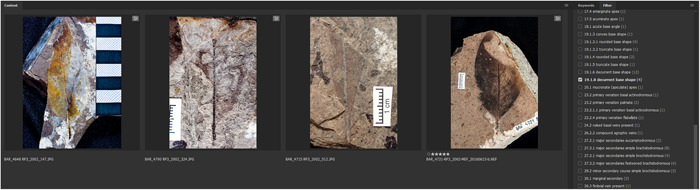
Using the filter tool.

**Figure A8:**
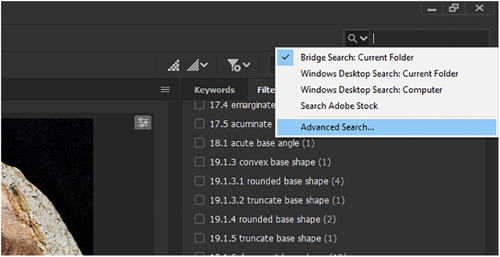
How to find the Advanced Search.

**Figure A9:**
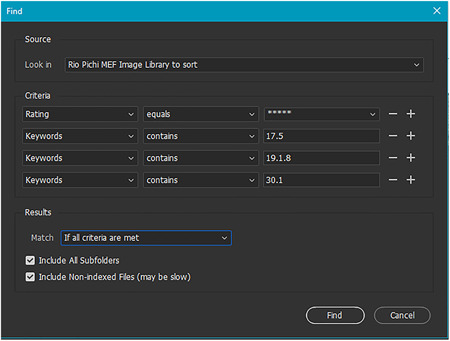
Searching by combinations of criteria.

**Figure A10:**
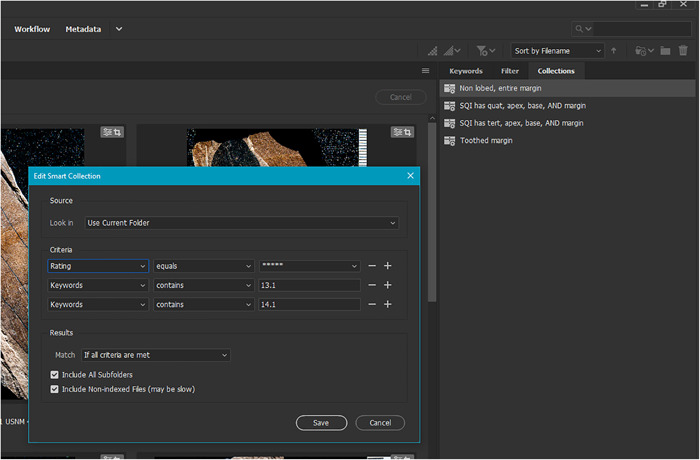
Opening and editing Smart Collections.

**Figure A11:**
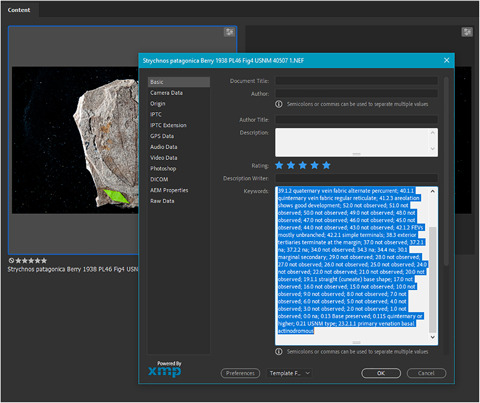
Viewing File Info to see assigned keyword metadata.

**Figure A12:**
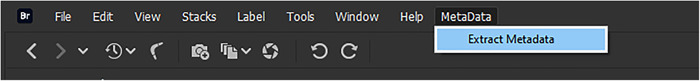
New Metadata toolbar created using the script provided in Appendix S2.

**Figure A13:**
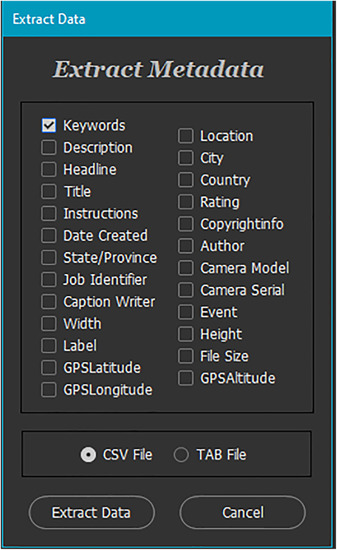
Extract Metadata dialog box.

## Data Availability

All necessary code is made available in Appendices S1–S3.

## Appendix 1 Detailed description of the workflow to use Adobe Bridge to assign character data to large libraries of specimen images and to use the provided scripts to export and reformat the Adobe Bridge keywords into character matrices.

I. Digital workspace set up
  a. Download and install Adobe Bridge (Adobe Inc., San Jose, California, USA). Bridge can be downloaded from the Creative Cloud desktop application with a free Adobe ID (https://creativecloud.adobe.com/apps/download/bridge) as a standalone item, without purchase. Bridge can be used with or without a paid subscription to the Adobe Creative Cloud, and it has continued functionality even after a free trial or a subscription ends. A paid subscription to the Adobe Creative Cloud provides access to the integrated Adobe Camera Raw application for reversible image adjustments and full integration with the other Creative Cloud applications, such as Photoshop.
  b. Upon opening Bridge, there are three resizable panes within the window. The standard configuration will be briefly described below (also note, by right‐clicking, panels can be opened or closed in any of the three panes, and these can be moved by tab‐dragging to customize the workspace fully).
    i. The Folders and Favorites panel is on the left by default, and is where the directory shortcut for the image library is set (see below).
    ii. At the top of the screen, there are several different view options to choose from, including “Essentials”, “Libraries”, “Filmstrip,” and others. The default view is Essentials, which displays the selected folder's content as thumbnails, whose size can be controlled using a slider. The Libraries and Filmstrip views allow for larger images and viewing side by side. The Metadata view shows smaller thumbnails (faster loading) and text attributes such as keywords.
    iii. On the right, we suggest closing the default “Preview” and “Publish” panels and opening the “Filter”, “Keywords”, and “Collections” panels. (Right‐click to select, or Window > Filter, Window > Keywords, Window > Collections).
  c. Image file organization
    i. Save all the image files to be compared in the same folder (i.e., using Windows Explorer) and take note of the folder's location.
    ii. We suggest using a standardized file naming system and format across image libraries for ease of alpha‐sorting by taxon or catalog number. The image file name will become the row name in the character matrix. Use the “Batch rename” tool in Bridge to quickly rename files or sets of selected files according to a customizable template (Tools > Batch rename OR right‐click, “Batch rename”)
    Example 1 for identified and citeable specimens:
    Species name + citation + Repository acronym + specimen number
    Cassia patagonica Berry 1938 PL20 Fig. 10 USNM_PAL_ 40394b.NEF
    Example 2 for unidentified collections:
    Repository acronym + specimen number + field number
    BAR_4474 RP3_2002_079.JPG
  d. Viewing an image library in Bridge
    i. To open an image library in Bridge, using the Folders panel (default location on the left‐hand side of the Bridge window), navigate to and select the file folder where the images are saved, and all images within that folder will be displayed as thumbnails in the center Content panel. Right‐click the file in the Folders panel to add it to the Favorites panel for easy future access and image library switching.
    ii. The image thumbnail size can be changed using the zoom slider at the bottom of the workspace.
    iii. When an image is selected, the space bar will bring the image into a zoomable full‐screen mode for viewing.
    iv. To view images and compare them side by side, select them in the “Essentials” workspace, then click on the “Libraries” or “Filmstrip” workspace (Figure A1).
    v. Open multiple Bridge workspace windows to view folders at the same time or side by side, which works especially well with multiple monitors.
    vi. (Adobe Creative Cloud subscribers) Right‐click and select “Open in Camera Raw…” to apply reversible image adjustments, including cropping the image, increasing contrast, adjusting white balance, temperature, etc., to aid in image analysis or to prepare images for publication (Figure A2).

II. Keywording images
  a. Import and edit the keyword metadata dictionary
    i. Open the Keywords panel. In the Keywords panel, the options menu (three horizontal bars on the far right) has the choice “Import…” to directly import any text file containing the desired keyword set or “Clear and import…” to clear any existing or preset keywords while importing the new set (Figure A3).
      1. To use our keyword set of leaf architecture and insect damage terms, navigate to where Appendix S1 is saved and open to import. Keywords will be displayed hierarchically in the Keywords panel with empty checkboxes next to each.
      2. If desired, a customized keyword metadata dictionary can be prepared in a text editor prior to upload in Bridge, as we have done with Appendix S1.
    ii. Edit keywords at any time within the Bridge Keywords panel (Right‐click, “Rename” or “Delete”) or create new keywords manually (from the Options menu, or with right‐click, “New Keyword” or “New Sub Keyword”).
      1. Note that even if a keyword is deleted from the list, if an image was previously given this keyword, it remains written to the file as metadata until the checkbox is unchecked while the image(s) is selected.
      2. If a keyword list is edited, or images opened with assigned keywords not present or not matching the current list verbatim, Bridge will still display and place the keyword in the list in the Keywords panel in italics. To add a keyword shown in italics to the list, right‐click and choose “Make Persistent”.
    iii. To save any changes made to the keyword set in Bridge, create a backup, or share a keyword set with another user: from the Options menu in the Keywords panel, choose “Export…” to save the keyword set to a new text file.
  b. Apply keywords to images
    i. Select an image thumbnail in the central Content panel while viewing an image in the “Essentials” workspace. In the Keywords panel, click the box next to any number of desired keywords to apply a check in the box and assign the character states to the image (Figure A4).
    ii. Reverse keyword assignments by simply unchecking the box next to the character while the image(s) is selected.
    iii. To batch keyword, select multiple images in the same folder to be keyworded with the same character, then check the boxes next to the characters in the Keywords panel.
  c. Apply ratings or labels to images and folders
    i. When an image thumbnail (or a folder) is selected, an outline of five stars (or five dots) appears immediately above the file name (Figure A5).
    ii. This is the rating system, which also functions like a keyword. Clicking on these stars applies ratings to images or folders to instantly filter to help prioritize specimens, indicate confidence levels, or keep track of exemplar specimens that are fully coded. Star ratings can also be added or removed from selected images using the “Label” menu, or with keyboard shortcuts, by pressing the CTRL + number, 0 through 5 **(**⌘ + number on Mac).
    iii. Similarly, labels are accessed by right‐clicking on an image, and selecting “Label” (or the “Label” menu). This can apply five different labels with distinct color codes to an image or folder (Figure A6). Label names can be customized in Edit > Preferences > Labels.

III. Using keywords
  a. While a folder of images is open: in the Filter panel, check the box next to the character state of interest, and all images with that assignment will be instantly filtered from the folder (Figure A7). Multiple character selections will show all images with ANY of the selected characters (OR logic). For more complex searches, see Advanced Search, next.
  b. The “Advanced Search” function within the Find command in Bridge (located next to the search bar at the top right of the window) allows for filtering by conditional combinations of different characters (Figure A8, A9). It will search within a single folder, a folder and all subfolders, or the entire computer/directory.
  c. An advanced search result can also be saved in the Collections panel as a Smart Collection, allowing rapid recall of specific subsets of images that will update automatically if keywords are changed (Figure A10).
  d. To display a list of associated keywords separated by semicolons, right‐click any image and select “File info…” (Figure A11). Copy and paste the list into a word processor for formulating a quick specimen description, for example, by deleting the numbers and adding some descriptive words to put it into sentence format. Note that Bridge writes metadata to the image file in the order that keyword boxes were checked. That is why we used a hierarchical numbering system in front of each character state and created a script to re‐order the characters in R.
  e. Proceed to Appendix 1, part IV if planning to create a character matrix.
IV. Exporting metadata
  a. Install the “Extract MetaData” export menu option to Bridge.
    i. Download and unzip Appendix S2, the open source script “Extract Metadata” by Paul Riggott that is reproduced here from PS‐SCRIPTS (https://www.ps-scripts.com/viewtopic.php?f=72&t=24358&sid=6529114a378b6e37ff888bf74ba105cf) with permission.
    ii. In Bridge (Windows), go to Edit > Preferences > Startup scripts > Reveal my scripts (For Mac, Adobe Bridge > Preferences > Startup Scripts > Reveal my scripts).
    iii. Drag the unzipped Appendix S2 file into the new window that opened. Close the window, and close Bridge.
    iv. Restart Bridge and select “yes” to using the script on startup.
    v. The new menu option on the top menu bar called “Metadata” should now appear after the “Help” option (Figure A12).
  b. To extract keyworded character data
    i. While viewing in the Content panel, select all the keyworded images to be included as individuals in the character matrix, then in the top menu bar select Metadata > Extract Metadata. In the dialog window (Figure A13) that opens, select “Keywords” and “CSV File”, then select Extract Data.
    ii. A CSV file of the keywords for each image is saved to the Desktop with the following file name that is stamped with the time of the completed data extraction.
  i.e., Extract Metadata‐9_39_17AM.csv
V. Use RStudio and the code in Appendix S3 to reformat the exported Adobe Bridge keywords CSV file into character matrices.
  a. Download RStudio Desktop (open source) at https://www.rstudio.com/products/rstudio/download/
  b. If using a Windows computer, download and install RTools, as instructed here: https://cran.r-project.org/bin/windows/Rtools/ (this will help keep R and the packages used in the code up to date).
  c. Open the R script (Appendix S3) in RStudio (File > Open File). The R code is annotated, describing the action taken in each step; it can be altered to customize the final matrix format.
  d. Below, we summarize how the R code works to reformat the matrix. **Before running the code in RStudio as is, make sure to follow all the bolded instructions below, which require some user editing or action prior to executing the code**. Once code is edited, the user can highlight all the code and select “Run”, or copy and paste it into the Console and press Enter, to automatically run all lines of the code to produce the matrices.
    i. Lines 1–7 install the necessary packages and libraries.
    ii. Lines 10–13 describe four critical steps to set up the RStudio directory so that the code can function properly.
      1. **Create a new folder**.
      2. **Add the Extract Metadata (.csv file) exported from Adobe Bridge (from the Desktop) into the new folder**.
      3. **Add the Appendix S1: Metadata Dictionary (.txt file), or a customized keywords .txt file, to the same folder**. This hierarchical metadata dictionary file needs to contain the same set of keywords that were applied to the images in Adobe Bridge, so that RStudio can sort the data consistently. Keep this in mind when making changes to the keywords in Adobe Bridge.
        a. If the images to be included in the matrix had existing keywords previously assigned to their metadata (i.e., before using this method), these keywords MUST be incorporated into the the Metadata Dictionary file for a matrix to be generated successfully. For example, additional keywords could be added into the numbering system under the “Attributes” category. Be sure to save a new final version of the metadata dictionary text file reflecting this, so that RStudio will be able to successfully create the final matrices. If prior keywords do not need to be incorporated in the matrix, it may be easier to simply archive image copies, then remove all keywords on the working copies before applying our method.
      4. **Set the session working directory in RStudio to the new folder just created**.
        **Session** > **Set Working Directory** > **Choose Directory**
    iii. In line 17, import the Metadata Dictionary. **Make sure to edit the code in line 17 to the exact file name used**. The file will only be imported if the working directory is set in RStudio to the folder containing the .txt file (see ii.3). The imported file will be called “data” in the code.
      data < ‐ read.delim(file = **“A2 Fossil Leaf Metadata Dictionary for Adobe Bridge 11‐5‐21.txt”**, header = FALSE, sep = “\t”,)
    iv. In line 20, import.csv metadata extracted from Adobe Bridge. **Make sure to edit the code in line 21 to the exact file name (i.e., the timestamp) of the metadata export**. The file will only be imported if the working directory is set in RStudio to the folder containing the Extract Metadata.csv file (see ii.3). The imported file will be called “ogfile” in the code.
      ogfile < ‐ read.csv(**“Extract Metadata‐12_34_16PM.csv”**, header = FALSE, sep = “,”)
    v. Lines 22–33 format the row names and delimit the character data. To see an example of what each row looks like, type split[[1]] to see the first row.
    vi. Lines 35–52 use the Metadata Dictionary provided (named “data”) to determine the categories of data to be used for separate matrices. In the case of the Paleobotany Metadata Dictionary, this corresponds to Attribute, Leaf Architecture, and Insect Damage Types.
    vii. Lines 53–123 sort any Attribute metadata into a separate matrix (“atmatrix”), on the basis of beginning with a 0.
    viii. Line 126 exports the Attribute matrix to a CSV file.
    ix. Lines 128–292 will create the Leaf Architecture Character matrix (“leafarcmatrix”) with the dimensions and column names determined in the previous step. Then, it will transfer the character states into the matrix by matching the semantic numerals in each morphotype to the first number in the column, if it is coded for that character.
      For example:
      Species A: 1.1, 2.4, 5.6, 7.2 (each number separated by a comma represents the character, followed by the character state after the decimal point).
      Character name: 1. Leaf shape
      The code will go through the characters present in Species A, and if there is information for character 1, it will transfer the character state, in this case 1, to the matrix. If there is no information for a given character, it will leave that cell empty. If there is more than one state per character, it will transfer the multiple states separated by a comma.
    x. Line 296 exports the character matrix into a CSV file, named “LeafArch_matrix.csv”, to the same directory folder unless an alternative path is specified.
    xi. Lines 300–365 will create the damage type matrix (“damtmatrix”) and fill it with information corresponding to each morphotype following the same procedure as step vii.
    xii. Line 368 exports the damage type matrix into a CSV file, named “DamageType_matrix.csv”, to the same directory folder unless an alternative path is specified.
